# Supplementary material for: Scaling cross-tissue single-cell annotation models
Source: bioRxiv. 2023 Oct 10:2023.10.07.561331. Preprint. [Version 1] doi: 10.1101/2023.10.07.561331 (PMC10592700; doi:10.1101/2023.10.07.561331)
Supplement: 3 [file NIHPP2023.10.07.561331v1-supplement-3.pdf]

bioRxiv preprint doi: https://doi.org/10.1101/2023.10.07.561331; this version posted October 10, 2023. The copyright holder for this preprint (which was not certified by peer review) is the author/funder, who has granted bioRxiv a license to display the preprint in perpetuity. It is made available under aCC-BY 4.0 International license.

Supplements

Supp. Table 1: Classification performance of different models.

	F1-score (macro avg.)	Number of runs to calculate standard deviation
scTab (deep learning)	0.8295 ± 0.0007	5
XGBoost (boosted decision trees)	0.8127 ± 0.0005	5

bioRxiv preprint doi: https://doi.org/10.1101/2023.10.07.561331; this version posted October 10, 2023. The copyright holder for this preprint (which was not certified by peer review) is the author/funder, who has granted bioRxiv a license to display the preprint in perpetuity. It is made available under aCC-BY 4.0 International license.

MLP (deep learning)	0.7971 ± 0.0012	5
Linear	0.7848 ± 0.0001	4
CellTypist (training data subsampled to 1.5 Mio cells)	0.7304 ± 0.0015	4

Supp. Table 2: Performance of lung-specific versus cross-organ models evaluated on holdout test set subset to only lung-specific data.

	F1-score (macro avg.) on lung holdout data	Number of runs to calculate standard deviation
scTab (cross-organ)	0.7062 ± 0.0122	5
scTab (lung only)	0.7220 ± 0.0078	5
Linear (cross-organ)	0.5291 ± 0.0041	4
Linear (lung only)	0.7146 ± 0.0040	5

Supp. Table 3: Effect of data augmentation on loss and F1-score (macro avg.) on holdout test set.

	Neg. log-likelihood	F1-score (macro avg.)	Number of runs to calculate standard deviation
w. augmentation	0.659 ± 0.04	0.7841 ± 0.0030	4
wo. augmentation	0.797 ± 0.05	0.7755 ± 0.0020	4
P-value	0.0039	0.0016	4

Supp. Table 4: Classification performance of models with tuned versus default hyperparameters.

	F1-score (macro avg.) with default parameters	F1-score (macro avg.) with tuned parameters	Number of runs to calculate standard deviation
XGBoost	0.5855 ± 0.0112	0.8127 ± 0.0005	4
CellTypist	0.6258 ± 0.0036	0.7304 ± 0.0015	4

Supp. Table 5: Number of donors and cells per cell type and tissue combination.

See *supp_table_donors_and_cells_per_tissue+cell_type.csv*

Supp. Table 6: Number of shared tissues across individual cell types.

See *supp_table_number_of_tisses_for_each_cell_type.csv*

bioRxiv preprint doi: https://doi.org/10.1101/2023.10.07.561331; this version posted October 10, 2023. The copyright holder for this preprint (which was not certified by peer review) is the author/funder, who has granted bioRxiv a license to display the preprint in perpetuity. It is made available under aCC-BY 4.0 International license.

Supp. Table 7: Total variation that can be attributed to the cell type before and after data augmentation

	Total variation attributed to cell type and donor (R^2)
original/non-augmented data	0.189
augmented data	0.164

Supp. Figure 1: Data loading performance during model training (with data shuffling) and inference (without data shuffling).

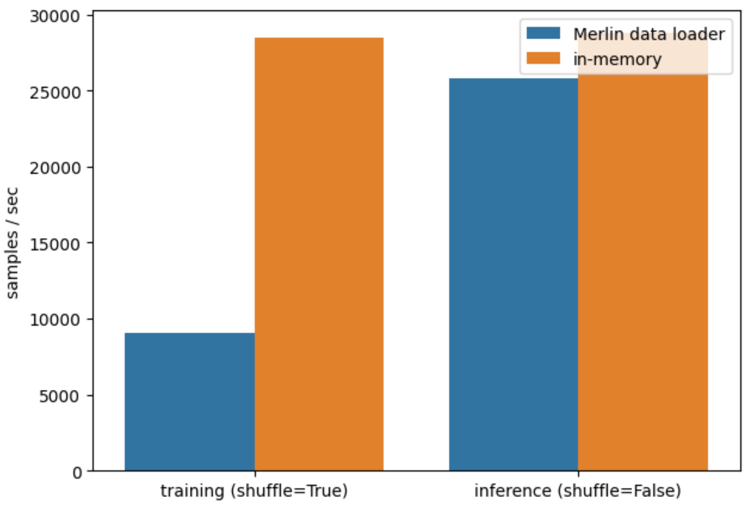

Benchmarks were run on a DGX-A100-320GB compute node with 14 cores and 80GB of memory allocated for the benchmark and half an A100 GPU (4g.20gb MIG). The training dataset consists of 15.2 million cells for the Merlin data loader and 1 million cells for the in-memory data loader. The validation dataset consists of 3.5 million cells for the Merlin data loader and 1 million cells for the in-memory data loader. Due to memory limitations for the in-memory data-loading, the training and validation set is subsampled to 1 million cells.

Supp. Figure 2: Number of shared tissues across individual cell types.

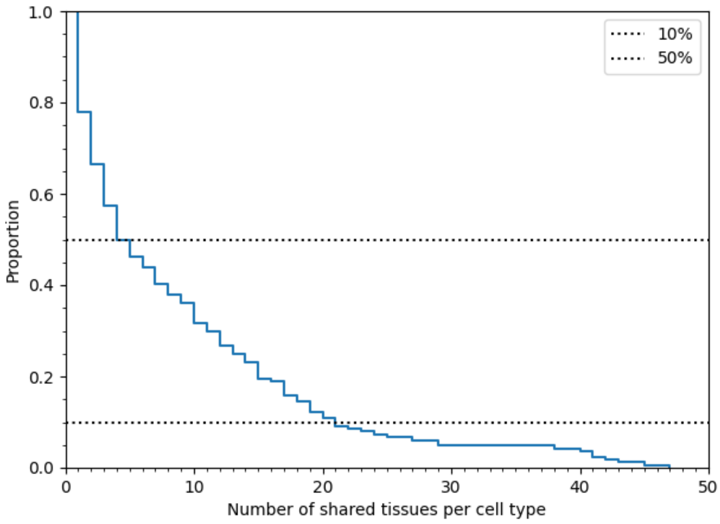

bioRxiv preprint doi: https://doi.org/10.1101/2023.10.07.561331; this version posted October 10, 2023. The copyright holder for this preprint (which was not certified by peer review) is the author/funder, who has granted bioRxiv a license to display the preprint in perpetuity. It is made available under aCC-BY 4.0 International license.

The complementary empirical cumulative distribution function of how many tissues each cell type is observed over (see Supp Table 7 for a per cell type statistic).

Supp. Figure 3: Learned features of scTab on holdout test data with granular cell type labels superimposed.

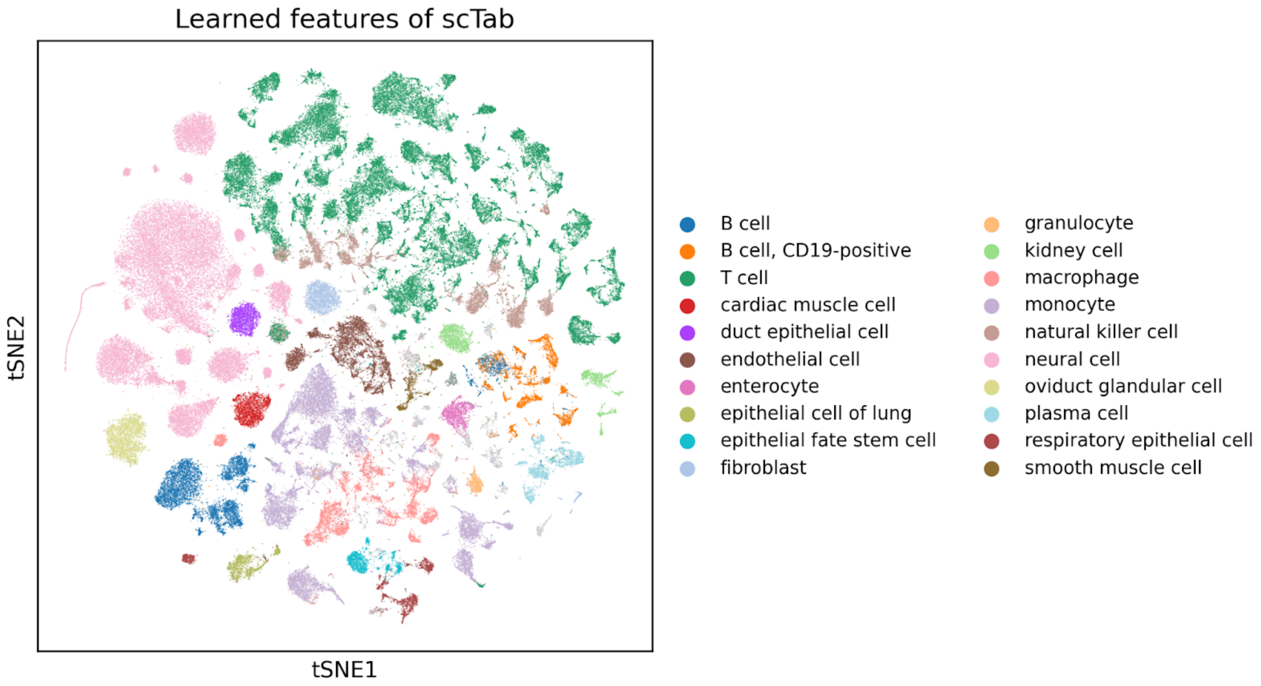

bioRxiv preprint doi: https://doi.org/10.1101/2023.10.07.561331; this version posted October 10, 2023. The copyright holder for this preprint (which was not certified by peer review) is the author/funder, who has granted bioRxiv a license to display the preprint in perpetuity. It is made available under aCC-BY 4.0 International license.

Supp. Figure 4: Learned features of scTab compared to the normalized gene expression of the input features on holdout test data subset to lung tissue only.

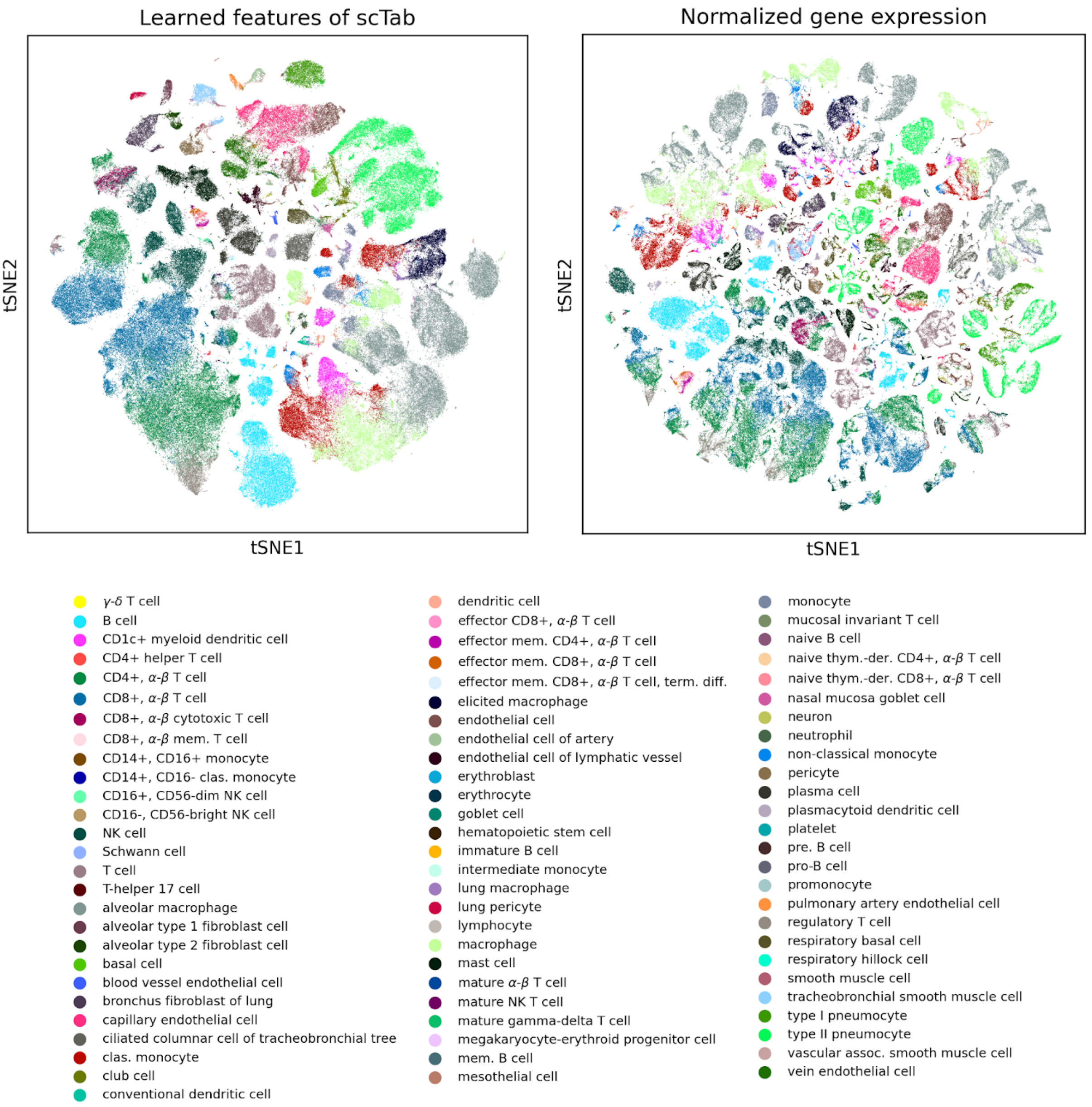

bioRxiv preprint doi: https://doi.org/10.1101/2023.10.07.561331; this version posted October 10, 2023. The copyright holder for this preprint (which was not certified by peer review) is the author/funder, who has granted bioRxiv a license to display the preprint in perpetuity. It is made available under aCC-BY 4.0 International license.

Supp. Figure 5: Uncertainty scores superimposed on tSNE plot of normalized gene expression on holdout test data.

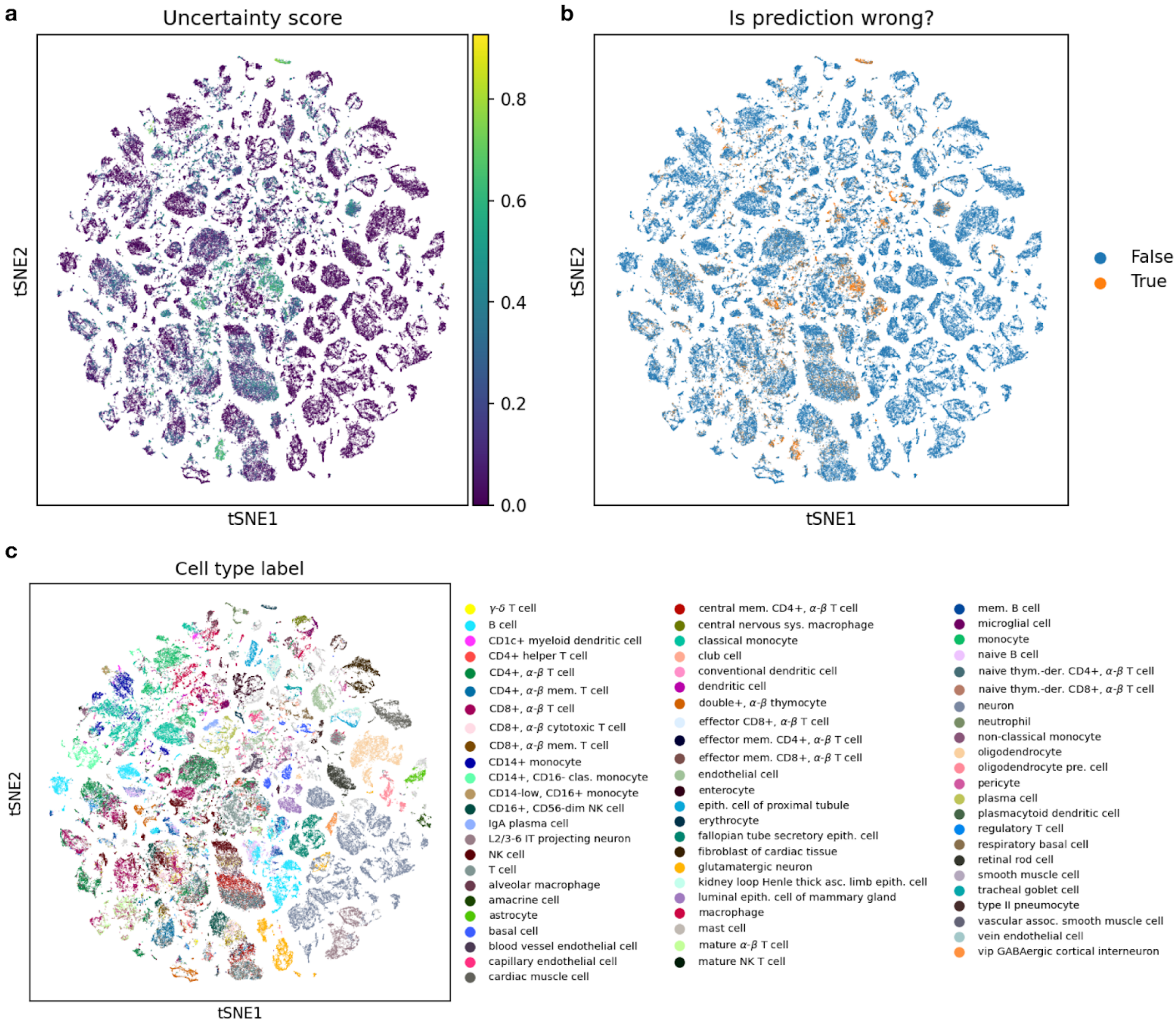

Uncertainty scores are calculated based on deep ensembles (averaged over 5 models) using $1 - maximum\, predicted\, probability$ to estimate the model uncertainty. All plots show tSNE embeddings of the normalized gene expression on the holdout test data. **(a)** Predicted uncertainty scores. **(b)** Binary indicator of whether a prediction was wrong to visually correlate uncertainty scores with wrong predictions. **(c)** Author annotated cell type labels for reference.
